# The Use of Harmonic Focus and Thunderbeat Open Fine Jaw in Thyroid Surgery: Experience of a High-Volume Center

**DOI:** 10.3390/jcm11113062

**Published:** 2022-05-29

**Authors:** Gian Luigi Canu, Fabio Medas, Federico Cappellacci, Francesco Casti, Raffaela Bura, Enrico Erdas, Pietro Giorgio Calò

**Affiliations:** Department of Surgical Sciences, University of Cagliari, Policlinico Duilio Casula, 09042 Monserrato, CA, Italy; fabiomedas@gmail.com (F.M.); fedcapp94@gmail.com (F.C.); frasticasti@gmail.com (F.C.); raffaela.bura@gmail.com (R.B.); erdasenrico@libero.it (E.E.); pgcalo@unica.it (P.G.C.)

**Keywords:** thyroid surgery, thyroidectomy, Harmonic Focus, Thunderbeat Open Fine Jaw, energy-based devices, complications

## Abstract

Background: In thyroid surgery, achieving accurate haemostasis is fundamental in order to avoid the occurrence of complications. Energy-based devices are currently extensively utilized in this field of surgery. This study aims to compare Harmonic Focus and Thunderbeat Open Fine Jaw with regard to surgical outcomes and complications. Methods: Patients submitted to total thyroidectomy in our center, between January 2017 and June 2020, were retrospectively analysed. Based on the energy-based device utilized, two groups were identified: Group A (Harmonic Focus) and Group B (Thunderbeat Open Fine Jaw). Results: A total of 527 patients were included: 409 in Group A and 118 in Group B. About surgical outcomes, the mean operative time was significantly shorter in Group B than in Group A (*p* < 0.001), while as regards complications, the occurrence of transient recurrent laryngeal nerve injury was significantly greater in Group B than in Group A (*p* = 0.019). Conclusions. Both Harmonic Focus and Thunderbeat Open Fine Jaw have proven to be effective devices. Operative times were significantly shorter in thyroidectomies performed with Thunderbeat Open Fine Jaw; however, the occurrence of transient recurrent laryngeal nerve injury was significantly greater in patients operated on with this device.

## 1. Introduction

Thyroidectomy represents the main operation in endocrine surgery. Although mortality in thyroid surgery is currently negligible, morbidity remains a challenging problem. Complications are mainly represented by hypoparathyroidism, cervical haematoma, and recurrent laryngeal nerve (RLN) injury [1,2,3,4,5,6,7,8,9,10,11].

Thyroid gland is a highly vascularized organ; therefore, achieving accurate haemostasis, which allows to prevent bleeding in the operating field, allowing an adequate view of the anatomic structures, is fundamental to avoid the occurrence of complications. Since thyroidectomy was standardized in the early 1900s by Emil Theodor Kocher, this surgical procedure has progressively developed. Clamp-and-tie technique, with monopolar or bipolar electrocautery, has traditionally been utilized to obtain haemostasis. Innovative energy-based devices were subsequently introduced to achieve the same purpose. These instruments, using different forms of energy, are currently widely utilized in this field of surgery. In particular, Harmonic Focus (HF; Ethicon, Johnson and Johnson, New Brunswick, NJ, USA) and Thunderbeat Open Fine Jaw (TB; Olympus, Tokyo, Japan) are two of the most commonly used devices [12].

HF works by means of ultrasonic energy. This device is able to coagulate and cut tissues simultaneously [1,13].

TB integrates advanced bipolar and ultrasonic energy. It is composed of a lower ultrasonic and bipolar probe and an upper bipolar jaw that allow two functional modes: “seal and cut” to coagulate and cut tissue simultaneously and “seal” for coagulation alone [1,13].

In the literature, several reports comparing the energy-based devices with the traditional technique exist. In most of these studies, Harmonic Focus and LigaSure Small Jaw (a device that works by means of advanced bipolar energy) [14,15,16,17,18,19,20,21,22,23] were evaluated. Far fewer studies that compare the different devices with each other exist [24,25,26,27,28,29,30,31]. Moreover, currently, about the use of Thunderbeat Open Fine Jaw in thyroid surgery, to our knowledge, in the literature, there are only five other clinical studies (in this field of surgery, other investigations involving this device were carried out on porcine models) [32,33,34,35,36,37,38,39]. 

This study aims to compare Harmonic Focus and Thunderbeat Open Fine Jaw with regard to surgical outcomes and complications.

## 2. Materials and Methods

### 2.1. Study Design

This study is based on a retrospective analysis regarding patients submitted to total thyroidectomy, between January 2017 and June 2020, in our Unit of General and Endocrine Surgery (University of Cagliari). 

Data were obtained from a prospectively maintained database.

We included in this study only conventional total thyroidectomies performed with HF and TB by the two most experienced thyroid surgeons of our center (who performed more than 100 thyroidectomies per year during the study period and were highly familiar with the use of both the devices). Among these patients, those simultaneously submitted to parathyroidectomy or lateral neck dissection and those with incomplete data were excluded from this study. 

Based on the energy-based device utilized, two groups were identified: patients submitted to thyroidectomy with HF were included in Group A, while those with TB in Group B.

Demographic data (sex, age), information about thyroid function (specifically as regards cases of hyperthyroidism), information regarding the surgical procedure (extent of surgery, use of intraoperative nerve monitoring), histological findings (thyroid weight, histological diagnosis), surgical outcomes (operative time, drain output on postoperative day 1, postoperative stay), and complications (hypoparathyroidism, recurrent laryngeal nerve injury, cervical haematoma, wound infection) were assessed. 

### 2.2. Endpoints

The primary endpoint was the occurrence of complications (recurrent laryngeal nerve injury, hypoparathyroidism, cervical haematoma, and wound infection), while secondary endpoints were operative time, drain output on postoperative day 1, and postoperative stay.

### 2.3. Surgical Procedure

All operations were performed under general anaesthesia. Central neck dissection was performed in patients with preoperative diagnosis or intraoperative suspicion of lymph node metastases. Recurrent laryngeal nerves and parathyroid glands were systematically searched and identified. To facilitate nerve identification and to confirm its functional integrity, intraoperative nerve monitoring (IONM) was almost always used. One or two closed suction drains were systematically placed below the strap muscles. The duration of the surgical procedure was estimated, in minutes, from skin incision to skin closure.

### 2.4. Postoperative Management 

Diagnosis of postsurgical hypoparathyroidism was made in the case of iPTH < 10 pg/mL following surgery (reference range: 10–65 pg/mL). Permanent hypoparathyroidism was diagnosed in the case of iPTH levels below the normal range for more than 6 months. 

Recurrent laryngeal nerve injury was diagnosed through postoperative fibrolaryngoscopy. After surgery, fibrolaryngoscopy was performed in all cases of suspected recurrent laryngeal nerve injury due to loss of signal at IONM or hoarseness. Recurrent laryngeal nerve lesion was defined as permanent in the case of vocal cord paralysis, identified through fibrolaryngoscopy, persisting for more than 6 months. 

### 2.5. Statistical Analysis

Statistical analyses were performed with MedCalc^®^ 19.1.3 (MedCalc Software Ltd, Ostend, Belgium). Chi-square test or Fisher exact test were used for categorical variables and *t*-test for continuous variables. *p*-values were considered statistically significant when <0.05.

## 3. Results 

Among the 1027 patients submitted to thyroid surgery in our center during the study period, 527 met the inclusion criteria: 151 males and 376 females, with a mean age of 53.64 ± 14.85 years. 

No statistically significant difference was found with regard to sex, age, cases of hyperthyroidism, extent of surgery, use of IONM, thyroid weight, and histopathological diagnosis between the two groups. These results are shown in Table 1.

Of the 527 patients enrolled, 409 were included in Group A and 118 in Group B.

In Group A, the average operative time was 87.35 ± 20.98 min, the average drain output on postoperative day 1 was 62.12 ± 23.38 mL, and the average postoperative stay was 2.93 ± 1.41 days. As regards complications, there were 83 (20.29%) cases of transient hypoparathyroidism, 18 (4.40%) cases of permanent hypoparathyroidism, 5 (1.22%) cervical haematomas, 7 (1.71%) unilateral RLN lesions, 1 (0.24%) bilateral RLN lesion, 5 (1.22%) transient RLN lesions, 4 (0.98%) permanent RLN lesions, and 3 (0.73%) wound infections.

In Group B, the average operative time was 74.47 ± 16.40 min, the average drain output on postoperative day 1 was 61.30 ± 23.19 mL, and the average postoperative stay was 3.02 ± 2.03 days. As regards complications, there were 22 (18.64%) cases of transient hypoparathyroidism, 7 (5.93%) cases of permanent hypoparathyroidism, 1 (0.85%) cervical haematoma, 5 (4.24%) unilateral RLN lesions, 1 (0.85%) bilateral RLN lesion, 6 (5.08%) transient RLN lesions, 1 (0.85%) permanent RLN lesion, and 1 (0.85%) wound infection.

No statistically significant difference was found in terms of drain output on postoperative day 1, postoperative stay, transient hypoparathyroidism, permanent hypoparathyroidism, cervical haematoma, unilateral RLN injury, bilateral RLN injury, permanent RLN injury, and wound infection between the two groups.

On the contrary, the mean operative time was significantly shorter in Group B than in Group A (*p* < 0.001), while occurrence of transient RLN injury was significantly greater in Group B than in Group A (*p* = 0.019).

Results regarding surgical outcomes and complications are shown in Table 2.

## 4. Discussion

Haemostasis has historically been considered a crucial point in thyroid surgery. During the 1800s, the mortality rate from thyroidectomy was approximately 40%. In the early 1900s, thyroid surgery was profoundly revolutionized by Emil Theodor Kocher. Through a precise surgical technique and accurate haemostasis, he was able to drastically reduce mortality and morbidity [1,12,13]. Currently, in order to obtain effective and safe haemostasis, surgeons have at their disposal energy-based devices, which work using different forms of energy [1,12,13].

This study aims to compare Harmonic Focus, which works by means of ultrasonic energy, and Thunderbeat Open Fine Jaw, which integrates advanced bipolar and ultrasonic energy, in terms of surgical outcomes and complications.

In order to limit bias related to surgeon experience, only operations performed by the two most skilled thyroid surgeons of our center, who performed more than 100 thyroidectomies per year during the study period, were considered. Moreover, these two surgeons were highly familiar with the use of both the energy-based devices.

In our study, the occurrence of transient hypoparathyroidism, permanent hypoparathyroidism, cervical haematoma, unilateral recurrent laryngeal nerve injury, bilateral recurrent laryngeal nerve injury, permanent recurrent laryngeal nerve injury, and wound infection was comparable between the two groups, while the occurrence of transient recurrent laryngeal nerve injury was significantly greater in patients operated on with TB than in those with HF. About surgical outcomes, we found a statistically significant difference in terms of operative times, which were, on average, shorter in operations performed with TB than in those with HF.

A few years ago, we performed a similar analysis comparing Harmonic Focus, Thunderbeat Open Fine Jaw, and LigaSure Small Jaw [32]. Furthermore, in this case, operative times were significantly shorter in thyroidectomies performed with TB than in those with HF, without any difference in terms of complications. However, this previous study was burdened by two important limitations: the huge numerical discrepancy between TB and HF groups (57 vs. 1012 patients, respectively) and the inhomogeneous utilization of the devices during the study period (while the use of HF covered the entire period, from 2012 to 2018, that of TB was restricted to the last years considered, from 2015 to 2018, thus benefiting from an increased experience of the surgeons). In the present study, differently, the groups are more balanced, the two devices are used homogeneously within the period considered, and moreover, the number of thyroidectomies performed with TB is considerably greater. Furthermore, differently from the previous analysis, in the present study, we evaluated whether recurrent laryngeal nerve lesions were transient or permanent. For these reasons, our current results may be considered more accurate.

As regards the other four clinical studies in which TB was considered, in all of these, no statistically significant difference was found in terms of complications between HF and TB [33,34,35,36]. However, in this regard, it is important to underline that Back et al. [34] reported a higher rate of transient recurrent laryngeal nerve injury in patients operated on with TB although the difference was not statistically significant (probably due to the limited size of their sample). Considering this finding, these authors recommend more caution when using this device near the recurrent laryngeal nerve. 

About operative times, only Back et al. and Papavramidis et al. described shorter operations in patients operated on with TB than in those with HF [34,35].

The result of our present analysis regarding the occurrence of transient recurrent laryngeal nerve injury confirms, also from a statistical point of view, that of Back et al. [34]. In our opinion, the greater occurrence of this complication in thyroidectomies performed with TB was due to a slightly greater lateral thermal spread generated by this device. However, it is important to underline that permanent recurrent laryngeal nerve lesions were comparable.

As regards operative times, shorter operations obtained using the energy-based devices rather than the traditional technique, as widely described in the literature [14,15,16,18,19,21,23], make it possible to increase the number of operations in the same surgical session, thus optimizing the utilization of the operating theatres and reducing the waiting lists. Furthermore, shorter operative times reduce anaesthesia times, allowing for faster recoveries after surgery, and decrease anaesthesia costs of each individual procedure. Ultimately, as regards the economic aspect, the gain in time leads to a decrease in the overall costs, which justifies the cost of the devices. Shorter operative times in thyroidectomies performed with TB compared with those with HF in our experience and in those of Back et al. and Papavramidis et al. [32,34,35], in our opinion, are due to a greater speed of TB in coagulation and cutting of tissues, probably resulting from the simultaneous use of two forms of energy.

Our study has two main limitations. Firstly, it is based on a retrospective analysis. However, in this regard, we want to specify that the choice of the energy-based device by the surgeon was generally random, sometimes simply related to their availability. The second limitation is represented by the fact that intraoperative nerve monitoring was not used in all patients. However, the percentage of patients undergoing surgery without the use of IONM is very low, and no statistically significant difference was found in this regard when comparing the two groups. Moreover, in these patients, postoperative fibrolaryngoscopy was always performed in the case of occurrence of hoarseness after surgery.

## 5. Conclusions

Both Harmonic Focus and Thunderbeat Open Fine Jaw have proven to be effective devices in the field of thyroid surgery. 

Operative times were significantly shorter in thyroidectomies performed with Thunderbeat Open Fine Jaw; however, the occurrence of transient recurrent laryngeal nerve injury was significantly greater in patients operated on with this device. Based on this last finding, we believe that more caution is needed near the recurrent laryngeal nerve when using Thunderbeat Open Fine Jaw.

Given the limitations of our analysis, other studies, possibly prospective and randomized, are needed in order to better compare these two energy-based devices.

## Figures and Tables

**Table 1:** Demographic data, information on thyroid function, information about the surgical procedure, and histological findings.

	Total (*n* = 527)	Group A (*n* = 409)	Group B (*n* = 118)	*p*-Value
Sex				
Male	151 (28.65%)	121 (29.58%)	30 (25.42%)	0.379
Female	376 (71.34%)	288 (70.42%)	88 (74.58%)	
Age				
(years, mean ± SD)	53.64 ± 14.85	53.55 ± 14.84	53.95 ± 14.93	0.796
Hyperthyroidism	162 (30.74%)	130 (31.78%)	32 (27.12%)	0.333
Extent of surgery				
TT	479 (90.89%)	372 (90.95%)	107 (90.68%)	0.310
TT + UCND	27 (5.13%)	23 (5.63%)	4 (3.39%)	
TT + BCND	21 (3.98%)	14 (3.42%)	7 (5.93%)	
Use of IONM	480 (91.08%)	374 (91.44%)	106 (89.83%)	0.588
Thyroid weight				
(g, mean ± SD)	53.74 ± 61.51	54.43 ± 63.32	51.34 ± 54.97	0.630
Histological diagnosis				
Benign disease	292 (55.41%)	224 (54.77%)	68 (57.63%)	0.582
Malignancy	235 (44.59%)	185 (45.23%)	50 (42.37%)	

SD, standard deviation; TT, total thyroidectomy; UCND, unilateral central neck dissection; BCND, bilateral central neck dissection; IONM, intraoperative nerve monitoring.

**Table 2:** Surgical outcomes and complications.

	Total (*n* = 527)	Group A (*n* = 409)	Group B (*n* = 118)	*p*-Value
Operative time				
(minutes, mean ± SD)	84.91 ± 20.54	87.35 ± 20.98	74.47 ± 16.40	<0.001
Drain output on POD 1				
(mL, mean ± SD)	61.91 ± 23.33	62.12 ± 23.38	61.30 ± 23.19	0.743
Postoperative stay				
(days, mean ± SD)	2.95 ± 1.57	2.93 ± 1.41	3.02 ± 2.03	0.614
Hypoparathyroidism				
Transient	105 (19.92%)	83 (20.29%)	22 (18.64%)	0.693
Permanent	25 (4.74%)	18 (4.40%)	7 (5.93%)	0.491
Cervical haematoma	6 (1.14%)	5 (1.22%)	1 (0.85%)	1.000
RLN injury				
Unilateral	12 (2.28%)	7 (1.71%)	5 (4.24%)	0.152
Bilateral	2 (0.38%)	1 (0.24%)	1 (0.85%)	0.398
Transient	11 (2.09%)	5 (1.22%)	6 (5.08%)	0.019
Permanent	5 (0.95%)	4 (0.98%)	1 (0.85%)	1.000
Wound infection	4 (0.76%)	3 (0.73%)	1 (0.85%)	1.000

SD, standard deviation; POD 1, postoperative day 1; RLN, recurrent laryngeal nerve.

## Data Availability

The data that support the findings of this study are available from the corresponding author upon reasonable request.
